# O:2-CRM_197_ Conjugates against *Salmonella* Paratyphi A

**DOI:** 10.1371/journal.pone.0047039

**Published:** 2012-11-07

**Authors:** Francesca Micoli, Simona Rondini, Massimiliano Gavini, Luisa Lanzilao, Donata Medaglini, Allan Saul, Laura B. Martin

**Affiliations:** 1 Novartis Vaccines Institute for Global Health, Siena, Italy; 2 Laboratorio di Microbiologia Molecolare e Biotecnologia (LA.M.M.B.), Dipartimento di Biotecnologie, Università di Siena, Siena, Italy; University of Western Ontario, Canada

## Abstract

Enteric fevers remain a common and serious disease, affecting mainly children and adolescents in developing countries. *Salmonella enterica* serovar Typhi was believed to cause most enteric fever episodes, but several recent reports have shown an increasing incidence of *S.* Paratyphi A, encouraging the development of a bivalent vaccine to protect against both serovars, especially considering that at present there is no vaccine against *S.* Paratyphi A. The O-specific polysaccharide (O:2) of *S.* Paratyphi A is a protective antigen and clinical data have previously demonstrated the potential of using O:2 conjugate vaccines. Here we describe a new conjugation chemistry to link O:2 and the carrier protein CRM_197_, using the terminus 3-deoxy-D-manno-octulosonic acid (KDO), thus leaving the O:2 chain unmodified. The new conjugates were tested in mice and compared with other O:2-antigen conjugates, synthesized adopting previously described methods that use CRM_197_ as carrier protein. The newly developed conjugation chemistry yielded immunogenic conjugates with strong serum bactericidal activity against *S.* Paratyphi A.

## Introduction

Enteric fever remains an important cause of morbidity and death in developing countries, and especially in Asia, high prevalence of severe disease is recorded in children and adolescents [1]. Enteric fever is caused by *Salmonella enterica* serovars Typhi (*S*. Typhi) and Paratyphi A (*S*. Paratyphi A). As far as it is known, in Africa and the Americas, enteric fever is almost exclusively caused by *S.* Typhi, however, in Asia a significant proportion is caused by *S.* Paratyphi A.

In Thailand, after the mass vaccination of school children with the crude heat-inactivated typhoid vaccine in 1977, a sharp decline of enteric fever incidence was recorded, together with a disproportionately higher number of cases of *S.* Paratyphi A [2]. A similar trend was recorded in China, where the introduction of Vi polysaccharide vaccine resulted in a marked decline in typhoid fever and coincided with a subtle increase in fever episodes caused by *S.* Paratyphi A [3]. Reports from India showed increasing incidence rates of enteric fever due to *S.* Paratyphi A in many regions; for instance, in New Delhi, *S.* Paratyphi A cases rose from 6.5% to 44.9% over a five-year period (1994–1998) [4]. Additional data from Pakistan, Bangladesh and Nepal indicate that cases of paratyphoid fever are increasing [5], [6], consistent with other reports from China, where *S.* Paratyphi A infection is responsible for up to 64% of enteric fever cases [7]–[10]. The apparent increase in *S.* Paratyphi A infections was also consistent with an increasing proportion of *S.* Paratyphi A infections found among returning travelers from endemic regions [11]–[13].

Additionally, the true prevalence of enteric fever caused by *S.* Paratyphi A is underestimated due to the lack of reliable diagnostic tools. Consequently, most cases of enteric fever are treated empirically without isolation and serotyping of the responsible organism [10]. Another concern in *S.* Paratyphi A-caused enteric fever is growing antibiotic resistance [14], [15] with a consequent increase of medical complications [12].

The emerging importance of *S.* Paratyphi A is of great concern, particularly because no vaccine is available. *S.* Paratyphi A and *S.* Typhi only infect humans and their infections are clinically indistinguishable. As a result, in areas with high incidence of paratyphoid fever there is the risk that even a highly effective *S.* Typhi vaccine may be perceived as being poorly efficacious. Thus, a combination vaccine covering both serotypes seems needed in Asia [16]. As a public health tool, addition of a *S.* Paratyphi A component to a *S.* Typhi vaccine has benefits beyond its direct impact on *S.* Paratyphi A.

Two vaccine types are licensed against typhoid fever, one based on the surface polysaccharide Vi and another based on the oral attenuated strain Ty21a. The unconjugated polysaccharide is ineffective in young children (licensed for children above 2 years old) and the Ty21a can only be used in children older than 5 years [17].

Attenuated *S.* Paratyphi A strains have been developed as live oral vaccines [18], [19]. In particular, CVD 1902 showed high serum IgG to both *S.* Paratyphi A O-polysaccharide and flagella antigens in preclinical studies [19] and a phase 1 study is currently ongoing.

The Novartis Vaccines Institute for Global Health (NVGH) is working on the development of a bivalent vaccine against both *Salmonella* serovars that cause enteric fever, by independent chemical conjugation of the Vi polysaccharide [20], [21] and the O-specific polysaccharide (O-antigen, OAg, O:2) of *S*. Paratyphi A to the carrier protein CRM_197_. In contrast to tetanus toxoid and diphtheria toxoid, other commonly used carrier proteins, CRM_197_ does not require chemical detoxification, so that homogeneous and consistent preparations of the purified antigen is readily obtained [22].Vi-CRM_197_ has shown promising results in Phase 1/2 studies in European adults [23] and is currently being evaluated in younger populations in endemic countries.

The *S.* Paratyphi A OAg was selected because it has been described as both an essential virulence factor and a protective antigen [24]. OAg alone is not immunogenic in mice, but when conjugated to tetanus toxoid as carrier protein can induce anti-LPS antibodies with bactericidal activity [24]. These OAg conjugates with tetanus toxoid were also shown to be safe and to elicit anti-OAg IgG antibodies in adults, teenagers, and 2- to 4-year-old children [25].

**Figure 1:**
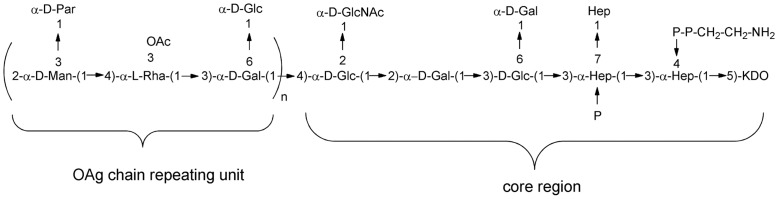
Structure of *S*. Paratyphi A O-antigen chain linked to the core region [22]; [23].

*Salmonella* lipopolysaccharide (LPS) consists of lipid A linked to the 3-deoxy-D-manno-octulosonic acid (KDO) terminus of a conserved core region, which is linked to a variable OAg chain. This serovar specific OAg chain is the immunodominant portion of the molecule. The OAg chain extends as a repeating polymer from the end of the core [26] and, in *S.* Paratyphi A, consists of a trisaccharide backbone composed of rhamnose (Rha), mannose (Man) and galactose (Gal), with a branch of paratose (Par) from the C-3 of mannose (which confers serogroup specificity: factor 2) and of glucose (Glc) from the C-6 of galactose (Figure 1) [24], [25]. C-3 of rhamnose is partially O-acetylated [27]. It has been reported that O-acetyl groups on the O-antigen chain of *S.* Paratyphi A are essential for conjugate immunogenicity [24].

Here we describe a new chemistry to create O:2-CRM_197_ conjugate vaccines. O:2 was extracted, performing acetic acid hydrolysis directly on the bacterial culture, without performing the step of LPS isolation. This acid treatment allows recovery from the supernatant of the OAg chain, with its O-acetylation level unchanged, linked to the core region (Figure 1), but with the toxic Lipid A removed. With OAg or O:2 we indicate this detoxified product having the KDO sugar at its reducing end, that has been used for conjugation.

The new conjugates were compared with other O:2-CRM_197_ conjugates synthesized according to previously published methods, and were used to immunize mice. Results of comparative immunogenicity and serum bactericidal activity are reported.

## Materials and Methods

### Reagents

The following chemicals were used in this study: 1-cyano-4-dimethylaminopyridinium tetrafluoroborate (CDAP), triethylamine (TEA), adipic acid dihydrazide (ADH), *N*-(3-dimethylaminopropyl)-*N*′-ethylcarbodiimide hydrochloride (EDAC), sodium hydrogen carbonate (NaHCO_3_), 4-morpholineethanesulfonic acid (MES), carbohydrazide (CDH), sodium cyanoborohydride (NaBH_3_CN), sodium acetate (AcONa), dimethyl sulfoxide (DMSO), ethyl acetate (AcOEt), dioxane, citric acid monohydrate, 6-aminohexanoic acid, O-(4-nitrobenzyl)hydroxylamine hydrochloride, sodium phosphate monobasic (NaH_2_PO_4_), phenol, 2,4,6-trinitrobenzenesulfonic acid solution 1 M in water (TNBS), pyrene butyric acid (PBA), pyridine, semicarbazide hydrochloride [Sigma]; sodium chloride (NaCl), sulfuric acid 96–98% (H_2_SO_4_) [Merck]; acetonitrile (CH_3_CN) [LC-MS Chromasolv]; sodium hydroxide (NaOH pellets) [Riedel-de Haën]; adipic acid bis(N-hydroxysuccinimmide) (SIDEA) [PFANSTIEHL Laboratories]; absolute ethanol [Carlo Erba]; sodium hydroxide (NaOH, 50% solution) [J. T. Baker] and sodium acetate salt (Pre-Weighed Reagent) [Dionex] for High-Performance Anion-Exchange Chromatography with Pulsed Amperometric Detection (HPAEC-PAD) analysis. D-(+)-Glucose monohydrate, D-(+)-Galactose, N-Acetyl-D-glucosamine [Sigma]; L-Rhamnose monohydrate, D-(+)-Mannose [Fluka] were used as monomer standards in the analysis.

Sephacryl S-300 HR was used for packing the column for conjugates purification; HiTrap™ desalting column 5 mL or HiPrep™ 26/10 desalting column 53 mL, prepacked with Sephadex™ G-25 Superfine, were used for samples desalting [GE Healthcare].

CRM_197_ was obtained from Novartis Vaccines and Diagnostics (NV&D).

### O-antigen Purification

*S*. Paratyphi A O-antigen was purified at NVGH after fermentation of the strain CVD1901, provided by the Center for Vaccine Development, University of Maryland.

Acid hydrolysis (1% acetic acid at 100°C for 6 hours) was performed directly on the fermentation culture and O-antigen was recovered in the supernatant by 0.22 µm microfiltration. Lower MW impurities were removed by tangential flow filtration (TFF), using a Hydrosart 30 kD membrane. Protein and nucleic acid impurities were co-precipitated in citrate buffer 20 mM at pH 3. Proteins were further reduced by ion exchange chromatography. Nucleic acids were further removed by precipitation adding 500 mM Na_2_HPO_4_, EtOH and 5 M CaCl_2_, to give a final concentration of 18 mM, 24% and 200 mM respectively, pH >4.5. OAg was then recovered in water by a second TFF 30 kD step. OAg recovery was high at each step of the process with an overall yield of around 80%, obtaining about 150 mg of purified OAg per liter of fermentation broth. All O-antigen preparations used for conjugates synthesis were characterized by protein content <1% (by micro BCA), nucleic acid content <0.5% (by A_260_), endotoxin level <0.1 UI/µg (by LAL), and O-acetyl content of 65–80% (by ^1^H NMR). Average molecular weight distribution was around 40–45 KDa, calculated based on the molar ratio Rha to N-acetyl glucosamine (GlcNAc) of 50–55 (by HPAEC-PAD analysis for sugar composition), considering that Rha is present per each OAg chain repeating unit and that GlcNAc is a unique sugar of the core region (Figure 1). GlcNAc quantification was in good agreement with KDO quantification by semicarbazide assay, confirming the presence of one α-chetoacid per OAg chain. OAg samples contained NH_2_ groups, as detected by TNBS [28] colorimetric method, probably as pyrophosphoethanolamine residues in the core region (Figure 1); NH_2_ amount was batch to batch variable and values from 15 to 43% (expressed as molar ratio % NH_2_ groups/GlcNAc) were found.

### Synthesis of O-antigen Conjugates

#### Random activation of the OAg chain with ADH by CDAP and conjugation with CRM_197_

This conjugate was synthesized based on the method reported by Konadu et al. [24], but using CRM_197_ as carrier protein. Thirty µL volume of CDAP (100 mg/mL in acetonitrile) was added per each mL of a solution 10 mg/mL of OAg in 150 mM NaCl, at room temperature (RT). The pH was maintained at 5.8 to 6.0 for 30 seconds, then 0.2 M TEA was added to reach pH 7.0 and the solution was mixed at RT for 2 minutes. Then 1 mL of 0.8 M ADH in 0.5 M NaHCO_3_ was added to 10 mg of OAg. The reaction was carried out for 2 h at RT, and the pH was maintained at 8.0 to 8.5 with 0.1 N NaOH. The reaction mixture was desalted using a G-25 column equilibrated with water and the product was designed as O:2-(CDAP)ADH. O:2-(CDAP)ADH was dissolved in MES 100 mM pH 5.8. An equal weight of protein was added, and the reaction mixture was put on ice (OAg to CRM_197_ ratio of 1∶1 in weight, with OAg concentration of 5 mg/mL). EDAC was added to a final concentration of 50 mM, and the reaction was mixed on ice for 4 h. The conjugate was designated as O:2-(CDAP)ADH-CRM_197_.

#### Activation of the terminus KDO with ADH (using the COOH group of KDO, by EDAC) and conjugation with CRM_197_ (by EDAC)

The OAg was solubilized in MES 100 mM pH 5.8 at a concentration of 3 mg/mL; ADH (ratio ADH to OAg of 1.36 by weight) was added before EDAC (EDAC final concentration of 3.7 mM). The reaction was mixed at RT for 4 h. The mixture was desalted using a G-25 column against water and the product was designed as O:2-(EDAC)ADH. The same procedure, used to obtain O:2-(CDAP)ADH-CRM_197_, was followed for the conjugation of O:2-(EDAC)ADH with CRM_197_. The conjugate was called O:2-(EDAC)ADH-CRM_197_.

#### New conjugation method: activation of the terminus KDO with ADH or CDH through its ketone group by reductive amination, followed by reaction of O:2-ADH or O:2-CDH with SIDEA and conjugation with CRM_197_ (Figure 2)

**Figure 2:**
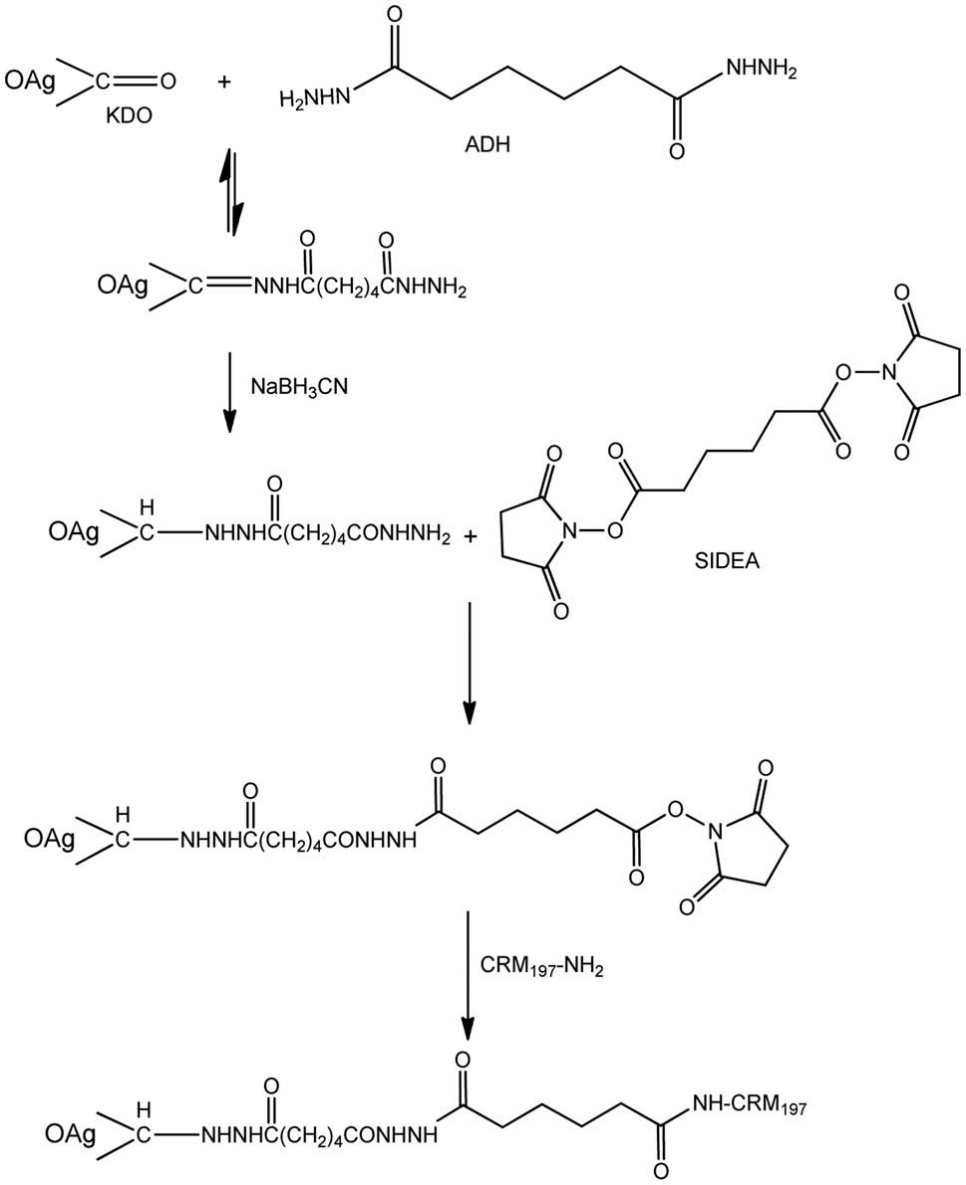
O:2-ADH-SIDEA-CRM_197_ conjugation scheme. Same reaction was also performed with the shorter linker CDH instead of ADH.

OAg was solubilized in 100 mM AcONa pH:4.5 at a concentration of 20–40 mg/mL. ADH (or CDH) then NaBH_3_CN were added as solids, both with a ratio 1.2∶1 by weight with respect to the OAg. The solution was mixed at 30°C for 1 h. The reaction mixture was desalted against water on a G-25 column and the derivatized product was designed as O:2-ADH or O:2-CDH.

For introduction of the second linker, SIDEA, 50 mg/mL O:2-ADH was dissolved in water/DMSO 1∶9 (v/v). When the polysaccharide was completely solubilized, TEA was added (molar ratio TEA/total NH_2_ groups = 5; total NH_2_ groups included both phosphoethanolamine groups on the OAg and the hydrazide groups introduced with the linker) and then SIDEA (molar ratio SIDEA/total NH_2_ groups = 12). The solution was mixed at RT for 3 h. Initial purifications were performed precipitating O:2-ADH-SIDEA by addition of AcOEt or dioxane (90% volume in the resulting solution) and then washing the pellet with the same organic solvent (ten times with 1/3 of the volume added for the precipitation) to remove free SIDEA. The purification process was subsequently modified to avoid use of toxic reagents as AcOEt or dioxane; the reaction mixture was added to a volume (equal to two times the reaction mixture volume) of 100 mM citrate pH 3 and mixed at 4°C for 30 min. Under these conditions, unreacted SIDEA precipitated and was separated by centrifugation. O:2-ADH-SIDEA was recovered from the supernatant by precipitation with EtOH (80% final). The pellet was washed twice with 1.5 volumes of 100% EtOH with respect to the reaction mixture volume and lyophilised. The product was designed as O:2-ADH-SIDEA. For conjugation to CRM_197_, O:2-ADH-SIDEA was solubilized in NaH_2_PO_4_ buffer pH 7.2 and CRM_197_ was added to give a protein concentration of 20 mg/mL, final buffer capacity of 100 mM and a molar ratio of active ester groups to CRM_197_ of 30 to 1. The reaction was mixed at RT for 2 h. The conjugate was designated as O:2-ADH-SIDEA-CRM_197_. A similar process was followed for generation of O:2-CDH-SIDEA-CRM_197_.

Direct derivatization of O:2 with SIDEA was also performed, using the same conditions as described above, but without previous modification with either ADH or CDH. A molar ratio of SIDEA to total NH_2_ groups present on the OAg itself of 12 was used. The resulting product was indicated as O:2-SIDEA. The same protocol described for O:2-ADH-SIDEA was used for conjugation of O:2-SIDEA with CRM_197_. The conjugate was designated as O:2-SIDEA-CRM_197_.

### Purification of the O-antigen Conjugates

All the conjugates were purified by size exclusion chromatography on a 1.6×90 cm S-300 HR column eluted at 0.5 mL/min in 50 mM NaH_2_PO_4_, 150 mM NaCl, pH 7.2.

### Analysis

Phenol sulfuric assay was used for total sugar content, using Glc as standard [29]. Micro BCA was used for total protein content, with bovine serum albumin (BSA) as a reference, following the manufacturer’s instructions [Thermo Scientific].

TNBS colorimetric method [28], [30] was used for total NH_2_ groups quantification. 6-aminohexanoic acid was used as standard for NH_2_ quantification on underivatized OAg samples, while ADH or CDH were used as standards for NH_2_ quantification after OAg derivatization with these linkers.

Random activation with ADH by CDAP was expressed as moles of linked ADH per mole of GlcNAc, indicating the average number of linkers introduced per OAg chain. Selective activation of the terminus KDO was calculated as moles of linked ADH or CDH per mole of GlcNAc %, indicating the % of activated OAg chains. Total NH_2_ groups were corrected by subtracting the number of NH_2_ groups already present on the un-derivatized OAg sample and the number of free NH_2_ groups (only for ADH), detected as free linker by RP-HPLC.

#### Sodium dodecyl sulfate-polyacrylamide gel electrophoresis (SDS-PAGE)

Conjugation mixtures were analyzed by SDS-PAGE using 7% Tris-acetate gels (NuPAGE, Invitrogen) to verify conjugate formation before performing purification. Samples (5–20 µL with a protein content of 5–10 µg) were mixed with 0.5 M dithiothreitol (1/5 v/v), NuPAGE LDS sample buffer (1/5 v/v) and heated at 100°C for 1 min. The gel, containing loaded samples, was electrophoresed at 45 mA in NuPAGE Tris-Acetate SDS running buffer (20x, Invitrogen) and stained with Simply Blue Safe Stain (Invitrogen).

#### High Performance Liquid Chromatography-Size Exclusion Chromatography (HPLC-SEC)

HPLC-SEC analysis was used to characterize conjugates, in comparison with free OAg and free CRM_197_. All samples were eluted on a TSK gel 6000 PW (30 cm×7.5 mm) column (particle size 17 µm; Sigma 8-05765) connected in series with a TSK gel 5000 PW (30 cm×7.5 mm) column (particle size 17 µm; Sigma 8-05764) with TSK gel PWH guard column (7.5 mm ID×7.5 cm L; particle size 13 µm; Sigma 8-06732) (Tosoh Bioscience). The use of the two columns in series gave better separation of conjugate from free saccharide and protein. Derivatized OAg samples were also characterized on a TSK gel G3000 PW_XL_ column (30 cm×7.8 mm; particle size 7 µm; cod. 808021) with TSK gel PW_XL_ guard column (4.0 cm×6.0 mm; particle size 12 µm; cod. 808033) (TosohBioscience), to verify that no cross-linked or degradation products were formed. The mobile phase was 0.1 M NaCl, 0.1 M NaH_2_PO_4_, 5% CH_3_CN, pH 7.2 at the flow rate of 0.5 mL/min (isocratic method for 60 min on TSK gel 6000+5000 and for 30 min on TSK gel G3000). Void and bed volume calibration was performed with □-DNA (□-DNA Molecular Weight Marker III 0.12–21.2 Kbp, Roche) and sodium azide (NaN_3_, Merck), respectively. O-antigen peaks were detected by differential refractive index (dRI), while UV detection at 214 nm and 280 nm was used for free protein and conjugates detection. Protein and conjugate peaks were also detected using tryptophan fluorescence (emission spectrum at 336 nm, with excitation wavelength at 280 nm).

For Kd determination, the following equation was used: Kd = (Te-T0)/(Tt-T0) where: Te = elution time of the analyte, T0 = elution time of the biggest fragment of ?-DNA and Tt = elution time of NaN_3_.

HPLC-SEC was also used to estimate the amount of unconjugated protein in conjugate samples. The area of unreacted CRM_197_ was quantified with respect to a calibration curve built with CRM_197_ samples in the range 5–50 µg/mL. The percentage of unconjugated CRM_197_ was calculated dividing the amount of free CRM_197_ detected by HPLC-SEC by the total amount of protein quantified in the sample by micro BCA.

#### High-Performance Anion-Exchange Chromatography with Pulsed Amperometric Detection (HPAEC-PAD)

Rha, Gal, Glc and Man, sugars of the OAg chain repeating unit, and GlcNAc, present as unique sugar in the core region, were estimated by HPAEC-PAD after acid hydrolysis of the OAg in free and conjugate samples to release the monosaccharides. Commercial monomer sugars were used for building the calibration curves. Paratose (Par), the other monosaccharide present in the OAg chain, could not be determined by this method as no commercially available standard exists, therefore the presence and amount of Par was determined by ^1^H NMR. For Rha, Gal, Glc and Man quantification, OAg samples, diluted to have the sugar monomer in the range 0.5–10 µg/mL, were hydrolyzed at 100°C for 4 h in 2 M TFA. This hydrolysis condition was optimal for release of all monomers without their degradation.

For GlcNAc quantification, OAg samples, diluted to have a GlcNAc concentration in the range 0.5–10 µg/mL, were hydrolyzed at 100°C for 6 h in 1 M TFA. After the hydrolysis, samples were chilled at 2–8°C for about 30 min, dried by SpeedVac overnight, reconstituted in water and filtered using 0.45 µm Acrodisc (PALL) filters before chromatographic analysis.

HPAEC-PAD was performed with a Dionex ICS3000 equipped with a CarboPac PA10 column (4×250 mm) coupled with PA10 guard column (4×50 mm). Separation of the sugars was performed with a flow rate of 1 mL/min eluting in a gradient from 10 mM NaOH to 18 mM NaOH over 20 min. After washing for 20 min with 100 mM AcONa in 28 mM NaOH, the column was re-equilibrated with 10 mM NaOH for 20 min.

The effluent was monitored using an electrochemical detector in the pulse amperometric mode with a gold working electrode and an Ag/AgCl reference electrode. The Dionex standard quadruple-potential waveform for carbohydrates was used. The resulting chromatographic data were processed using Chromeleon software 6.8. Calibration curves were built for each sugar monomer (0.5–10 µg/mL). The standards were hydrolysed and analysed in the same way as samples. For GlcNAc, glucosamine (GlcNH) was the species detected by HPAEC-PAD after hydrolysis.

#### KDO quantification by semicarbazide coupled with HPLC-SEC/

KDO was quantified by HPLC-SEC after derivatization with semicarbazide. Derivatization was performed slightly modifying the semicarbazide assay for α-chetoacids determination [31].

OAg samples and KDO standards (100 µL of total volume in water), with a C = O concentration between 15.7 nmol/mL and 156.7 nmol/mL, were added of 100 µL of semicarbazide solution (100 mg semicarbazide hydrochloride +90.5 mg of sodium acetate anhydrous in 10 mL of water). Blanks of the samples were also prepared: 100 µL of the OAg samples at the same concentration than for the analysis were added with 100 µL of a solution containing only sodium acetate (90.5 mg of sodium acetate anhydrous in 10 mL of water). All the samples and the standards were heated at 50°C for 50 minutes and then analyzed by HPLC-SEC (80 µL injected), on a TSK gel G3000 PW_XL_ column with guard column in 0.1 M NaCl, 0.1 M NaH_2_PO_4_, 5% CH_3_CN, pH 7.2 at the flow rate of 0.5 mL/min (isocratic method for 30 min). Detection was fixed at 252 nm. Area of the peak corresponding to the OAg after derivatization with semicarbazide was corrected with the area of the corresponding blank and the amount of KDO, attached to the OAg, calculated with the calibration curve built with the areas at 252 nm of KDO standards.

#### ^1^H NMR spectroscopy

Nuclear Magnetic Resonance (NMR) analysis was performed to estimate the O-acetylation level. It was also used as a confirmation of the identity of the OAg samples (typical signals of the OAg chain can be detected, confirming the presence of the characteristic sugars) and in particular for calculating the molar ratio of Rha to Par by comparing the integrals of the two peaks corresponding to Rha-H6 and Par-H6, at 1.40 and 1.28 ppm respectively. NMR analysis was also used to detect the presence of organic impurities.

Dried OAg samples and conjugates were subsequently solubilized in deuterium oxide (D_2_O) and transferred to 5 mm NMR tubes. For every sample, two ^1^H NMR spectra were collected: the first one in D_2_O and the second one after de-O-acetylation achieved by adding sodium deuteroxide (NaOD) to a final 200 mM concentration and heat treatment (37°C for 2 h for complete de-O-acetylation). The first ^1^H NMR spectrum was recorded to ensure the absence of impurities at the same chemical shift of the acetate anion released after de-O-acetylation of the sample that would interfere with the quantification of the O-acetyl content. O-acetylation level was quantified by comparing acetate (released after treatment with NaOD, at 1.91 ppm) and Rha-H6 peaks, and expressed as molar % of O-acetyl with respect to OAg chain repeating units (based on Rha present only in the OAg chain at one sugar per repeating unit).

NMR experiments were recorded at 25°C on Varian VNMRS-500 spectrometer, equipped with a Pentaprobe. Acquisition time of 5 sec, relaxation delay of 15 sec and number of scans of 64 were set for the acquisition of the spectra. For data acquisition and processing VNMRJ ver. 2.2 rev. C and Mestrenova 6.1 (Mestrelab Research) were used respectively. 1-D proton NMR spectra were collected using a standard one-pulse experiment. Chemical shifts were referenced to the free acetate anion at 1.91 ppm.

#### Free ADH quantification by Reversed Phase-High Performance Liquid Chromatography (RP-HPLC)

Amount of free ADH was calculated by RP-HPLC analysis of the samples after reaction of derivatization with PBA. One hundred µL of OAg samples derivatized with ADH, in water, were added to 300 µL of a solution prepared mixing 4∶1:1 in volume respectively of 2.5 mM PBA in DMSO, 20% v/v pyridine in DMSO and 2 M EDAC in water. Samples were heated at 40°C for 60 min. After heating, samples were dried in SpeedVac at 60°C overnight, solubilized in 400 µL of the mobile phase and passed through 0.2 nylon filters before analysis. The calibration curve was built with 0.024–0.4 µg/mL ADH and these standards treated as the samples.

Samples and standards were run on a Kinetex C18 column (Phenomenex, 2.6 µm 100A 150×4.6 mm, cod. 00F-4462-E0), eluting at 1 mL/min with 65% CH_3_CN in water under isocratic conditions for 10 min. The column was then washed for 15 min with 95% CH_3_CN and equilibrated again with 65% CH_3_CN for 20 min. Fluorescence detector was set with excitation wavelength at 345 nm and emission wavelength at 480 nm to see only the emission of the di-derivatized PBA-ADH-PBA and not the reactant PBA [32]. Percentage of free NH_2_ groups was calculated as molar ratio % of free ADH×2 divided by total NH_2_ groups introduced after derivatization with ADH (quantified by TNBS).

#### Total active ester groups quantification by A_260_

OAg sample derivatized with SIDEA was solubilized in water to a sugar concentration of 5 mg/mL. Immediately after solubilization, 250 µL of this solution were added to 500 µL of water and A_260_ was immediately measured (as blank). Another 250 µL of the OAg solution were added to 500 µL 0.1 M NH_4_OH to release the N-hydroxysuccinimide groups and the absorption of the N-hydroxysuccinimidate anion was measured at 260 nm. The calibration curve was built with 20–200 nmol/mL N-hydroxysuccinimide [33]. Percentage of derivatization with SIDEA was calculated as % molar ratio of linked active ester groups (subtracting the moles of free active ester groups quantified by RP-HPLC) to total NH_2_ groups before derivatization measured by TNBS. The ratio indicates the % in moles of NH_2_ groups activated with this reaction.

#### Total active ester groups quantification by ^1^H NMR after derivatization with O-(4-Nitrobenzyl)hydroxylamine

The introduction of active ester groups on the OAg chain was verified by ^1^H NMR, after sample derivatization with O-(4-Nitrobenzyl)hydroxylamine. Ten milligrams of the OAg sample derivatized with SIDEA were added to 72 µL of a 7 mg/mL solution of the reagent in EtOH-NaH_2_PO_4_ 200 mM pH 7.2 (1∶11 v/v) and mixed at RT for 1 h. After this time the mixture was diluted to 1 mL with water and de-salted on a G-25 column against water. The void volume was dried in SpeedVac and analyzed by ^1^H NMR. Typical aromatic signals of the O-(4-Nitrobenzyl) hydroxylamine were detected at 7.72 and 8.32 ppm and compared with Rha-H6 peak for the quantification.

#### Free SIDEA quantification by RP-HPLC

OAg samples derivatized with SIDEA were solubilized in 50% CH_3_CN in water at concentrations of 5–10 mg/mL total sugar. Calibration curve was built with 0.5–5.0 µg/mL SIDEA in 50% CH_3_CN. Standards and samples were filtered through 0.2 nylon filters and run on a Kinetex C18 column (Phenomenex, 2.6 µm 100A 150×4.6 mm, cod. 00F-4462-E0) with a flow rate of 1 mL/min eluting with 50% CH_3_CN in isocratic condition for 10 minutes. Detection was set at 195 nm. Percentage of free active ester groups was calculated as molar ratio % of free SIDEA×2 divided by total active ester groups (determined by A_260_ after ammonia treatment).

### Ethics Statement

All animal experimental work was approved by the Italian Animal Ethical Committee.

### Immunogenicity Study in Mice

Female 5 weeks old outbred CD-1 mice were purchased from Charles River Laboratories (Wilmington, MA) and maintained at Novartis Vaccines and Diagnostics. Eight mice per group were injected subcutaneously three times, at 2 week intervals, with 200 µL/dose of either 1 or 8 µg of O:2, as detailed in Table 1. Mice were bleed and sera collected before first immunization (day 0), on immunization days 14 and 28 and again 2 weeks after the third immunization, on day 42.

**Table 1:** Reductive amination of O:2-KDO with ADH is pH dependent and temperature independent.

Buffer	Temperature	% activated O:2
Sodium acetate pH 4.5	30°C	65.8
Sodium acetate pH 4.5	50°C	65.4
Sodium acetate pH 4.5	60°C	68.3
MES pH 6.0	30°C	43.9
Phosphate pH 8.0	30°C	26.2

### Serum Antibody Analysis by ELISA

Serum IgG levels against both O:2 and CRM_197_ were measured by ELISA using a previously described ELISA method [20], [21], with the following modifications. Wells of 96-well ELISA plates (Maxisorp, Nunc) were coated with 100 µL of 15 µg/mL O:2 (or 2 µg/mL CRM_197_) in 0.05 M carbonate buffer, pH 9.6. Mouse sera were diluted 1∶200 in PBS containing 0.05% Tween 20 and 0.1% BSA. ELISA units were expressed relative to mouse anti-O:2 or anti-CRM_197_ standard serum curves, with best 4 parameter fit determined by modified Hill Plot. One ELISA unit was defined as the reciprocal of the standard serum dilution that gives an absorbance value equal to 1 in this assay. Each mouse serum was run in triplicate. Data are presented as scatter plots of individual mouse ELISA units, and geometric mean of each group.

To detect anti-O:2 subclass IgG antibodies, day 28 serum samples from mice immunized with 8 µg/dose of OAg conjugates and unconjugated OAg were two-fold diluted (starting from 1∶40). The ELISA was carried out similarly to total IgG except that alkaline phosphatase labelled goat anti-mouse IgG1, IgG2a or IgG3 (1∶1500, Southern Biotechnology) were used as secondary antibodies. Plates were read at 405 nm using a 340 ATC reader (SLT Labinstument). Concentrations of OAg-specific IgG subclasses were calculated against a standard curve of mouse myeloma IgG1, IgG2a or IgG3 (Southern Biotechnology) determined on the same plate.

### Serum Bactericidal Activity (SBA)

*S.* Paratyphi A CVD1901 was grown in Luria Bertani (LB) medium to log-phase (OD: 0.2), diluted 1∶15,000 in SBA buffer (50 mM phosphate; 0.041% MgCl_2_ 6H_2_O; 33 mg/mL CaCl_2_; 0.5% BSA) to approximately 1.8×10^3^ colony forming units (CFU)/mL and distributed into sterile polystyrene U bottom 96-well microtiter plates (12.5 µL/well). To each well (final volume 50 µL, ∼450 CFU/mL), pooled sera samples serially diluted 1∶3 (starting from 1∶20) were added. Pooled sera from the different immunization groups were heated at 56°C for 30 min to inactivate endogenous complement. Active Baby Rabbit Complement (BRC, Pel-Freez Biologicals 04) used at 20% of the final volume was added to each well. BRC source, lot and percentage used in SBA reaction mixture was previously selected by lowest toxicity against CVD1901. The anti-O:2 ELISA standard serum plus active BRC was included as positive control. To evaluate possible nonspecific inhibitory effects of complement, bacteria were also incubated with anti-O:2 ELISA standard serum plus heat-inactivated BRC (iBRC). To evaluate possible inhibitory effects of mouse sera, bacteria were also incubated in SBA buffer without sera and active BRC (negative control). Each sample and control was tested in triplicate. Ten microliter reaction mixture from each well was spread on agar-plates at time zero to assess initial colony forming units (CFU), and at 2 h after incubation at 37°C. Plates were incubated overnight at 37°C and resulting CFU were counted the following day. Bactericidal activity was determined as percent CFU of test sera dilution with active (or inactive) BRC, compared with CFU of negative control.

### Statistical Analysis

Statistical analysis of ELISA results was conducted on day 42 samples. Groups were compared using Kruskal-Wallis One-Way ANOVA. Post hoc analysis was performed using Student-Newman-Keuls test for both anti-O:2 and anti-CRM_197_ antibody units (using an α = 0.05).

## Results

### Reductive Amination between OAg KDO and ADH

The method used for performing OAg reductive amination through the ketone group of KDO was developed starting from the conditions reported for the semicarbazide assay for α-chetoacids determination [31]. Preliminary tests were done adding ADH to 20 mg/mL OAg in 100 mM sodium acetate at pH 4.5 (ADH/OAg ratio of 1.2 in weight) and mixing the reaction at 30°C for 1 h. After this time, the pH was increased to 7.5 and NaBH_3_CN was added (NaBH_3_CN/OAg ratio of 1.2 in weight). The mixture was left at 30°C for 1 h. Using these conditions, OAg activation was 20.0% (calculated as moles of linked ADH/moles GlcNAc×100). The same procedure was used increasing the reaction time of the first step to 2 h, but OAg derivatization did not improve. NaBH_3_CN addition was then performed without altering the pH (at pH 4.5), obtaining 59.7% of OAg activation. Higher activation levels (69.4%) were obtained adding NaBH_3_CN together with ADH and mixing the reaction for 1 h. Percentage of OAg activation did not increase if the reaction time was prolonged to 2 h (65.9%). Results were reproducible when repeated.

Results obtained testing the effect of different pH and different temperatures are shown in Table 1; in all cases, ADH and NaBH_3_CN were added at the same time and the solution was mixed for 1 h. The best activation was obtained at lower pH and was temperature independent.

The possible use of less expensive NaBH_4_ instead of NaBH_3_CN was also evaluated. This was tested by simultaneous addition of ADH and the reducing agent (ADH/OAg and NaBH_4_/OAg ratios of 1.2 in weight) to 20 mg/mL O:2 in 100 mM NaOAc pH 4.5, and allowing the reaction to proceed for 1 h at 30°C. Only 30.7% of OAg chains were activated using NaBH_4_ compared to 65.8% when using NaBH_3_CN. This result was in line with higher NaBH_3_CN selectivity and stability at low pH compared to NaBH_4_ [34]. Optimized conditions for the reaction of O:2 with ADH were also applied to the synthesis of O:2-CDH.

### Conjugates Characterization

Using CRM_197_ as carrier protein, a novel conjugation method was developed (Figure 2) using ADH as linker and compared with two other methods already reported in the literature [24], [35]. The KDO at the end of the core region was derivatized with the linkers ADH and SIDEA and subsequently used for conjugation to the protein, without modifying the O-antigen chain. This method was compared with another selective method also using the terminus KDO, that resulted in the conjugate O:2-(EDAC)ADH-CRM_197_. Comparisons were also made to a conjugate prepared by random activation of the OAg chain, O:2-(CDAP)ADH-CRM_197_. The new chemistry was also applied to the use of CDH as linker to study the effect of a shorter linker than ADH on immunogenicity.

For each conjugation procedure, OAg intermediates were characterized for sugar recovery and % activation. In all cases, sugar yield post-desalting was >70%. Following precipitation, recoveries for O:2-ADH-SIDEA or O:2-CDH-SIDEA were >80%. O:2-(CDAP)ADH was characterized by 8–9 linkers introduced per OAg chain. O:2-(EDAC)ADH was characterized by 41% activation. For both O:2-ADH and O:2-CDH, activation ≥65% was obtained. The reaction with SIDEA resulted in ≥80% of NH_2_ groups modified in active ester groups.

O:2 derivatization, via ADH and SIDEA, was characterized by good reproducibility both in terms of sugar recovery and percentage of activation. Table 2 summarizes results from 5 different lots prepared at 300 mg scale for O:2-ADH and at 100 mg scale for O:2-ADH-SIDEA.

**Table 2:** Reproducibility of O:2-ADH and O:2-ADH-SIDEA intermediates.

Sample	% Sugar recovery(average ± SD)	% Activated O:2 chainwith ADH (average ±SD)	% Free/totalhydrazide groups	% Derivatized withSIDEA (average ± SD)	% Free/total active ester groups
O:2-ADH	76.0±4.4	80±0.4	<5	–	–
O:2-ADH-SIDEA	84.8±7.9	–	–	81.9±2.2	<10

Following activation with SIDEA, the number of active ester groups found by ^1^H NMR after derivatization of the sample with O-(4-Nitrobenzyl)hydroxylamine were in good agreement with values found by A_260_ after alkaline treatment. Residual NH_2_ groups detected by TNBS were ≤20%, with respect to the total amount quantified pre-activation with SIDEA.

**Figure 3:**
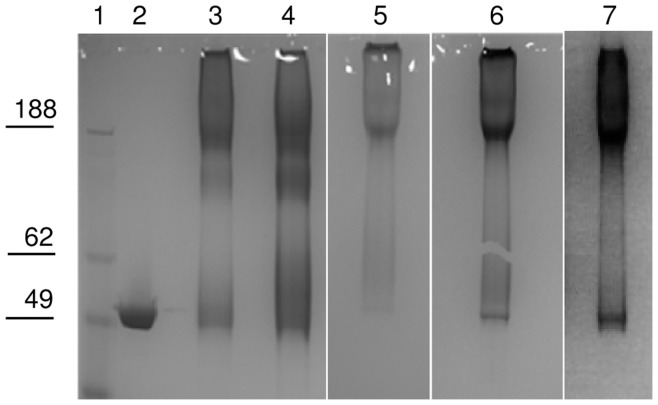
SDS-PAGE analysis of conjugation mixtures in comparison to unconjugated CRM_197_. Lane 1: marker, lane 2: CRM_197_, lane 3: O:2-(CDAP)ADH-CRM_197_ conjugation mixture, lane 4: O:2-(EDAC)ADH-CRM_197_ conjugation mixture, lane 5: O:2-ADH-SIDEA-CRM_197_ conjugation mixture, lane 6: O:2-CDH-SIDEA-CRM_197_ conjugation mixture, lane 7: O:2-SIDEA-CRM_197_ conjugation mixture. Ten µg of protein were loaded per conjugation mixture, 5 µg for CRM_197_.

All conjugation reaction mixtures were analyzed by SDS-PAGE before proceeding with the purification. SDS-PAGE analysis (Figure 3) of the reaction mixtures for the different conjugation methods tested showed traces of residual free protein in O:2-(CDAP)ADH-CRM_197_ (Lane 3) and more free protein in O:2-(EDAC)ADH-CRM_197_ (Lane 4). For O:2-ADH-SIDEA-CRM_197_ (Lane 5) and O:2-CDH-SIDEA-CRM_197_ (Lane 6) <10% of CRM_197_ remained unconjugated (estimated by HPLC-SEC) in reaction mixtures performing conjugation with a molar ratio of active ester groups to CRM_197_ of 30 and a protein concentration of 20 mg/mL. Furthermore for O:2-(CDAP)ADH-CRM_197_ and O:2-(EDAC)ADH-CRM_197_, analysis showed a diffuse free CRM_197_ band as monomer and the presence of CRM_197_ dimer. This suggests substantial modification or internal crosslinking of the free CRM_197_ and this presumably has almost certainly happened to the CRM_197_ that has linked to the O:2.

**Figure 4:**
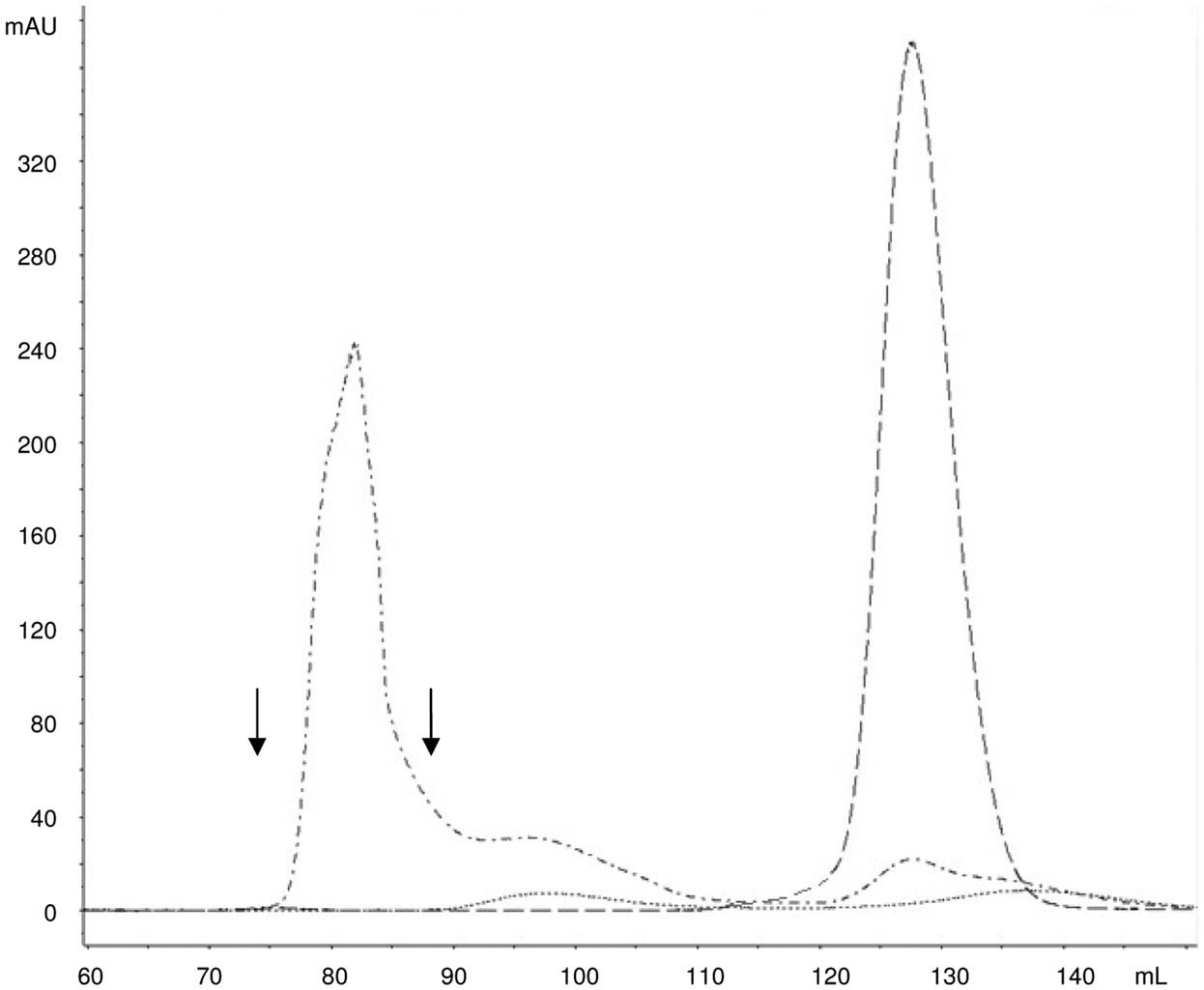
Gel filtration profiles on Sephacryl S-300 HR column (1.6×90 **cm) of O:2-ADH-SIDEA-CRM_197_ conjugation mixture (dash dot line; 880 µg of total protein injected; kd of 0.20), free O:2 (dotted line; 6.7 mg of sugar injected; kd of 0.33) and free CRM_197_ (dashed line; 1 mg of protein injected; kd of 0.61) at 214 nm.** Elution with 50 mM NaH_2_PO_4_, 0.15 M NaCl, pH 7.2 at a flow rate of 0.5 mL/min. Arrows indicate the fractions pooled to generate purified conjugate.

All conjugates were purified by size exclusion chromatography. A pool of purified higher molecular weight conjugate was collected that did not contain free saccharide and free protein, based on profiles of free O:2 and free CRM_197_ analyzed using the same column and the same eluting conditions (Figure 4). Conjugates were characterized (Table 3) prior to use in immunogenicity studies in mice. Conjugates synthesized by different chemistries (in particular random or selective chemistry) had a different O:2 to CRM_197_ ratio.

**Table 3:** Characterization of the conjugates used in the immunogenicity study in mice.

Conjugate	Total sugar, µg/mL	Total protein, µg/mL	wt/wt ratio O:2 to CRM_197_	Kd (HPLC-SEC)
O:2-(CDAP)ADH-CRM_197_	32.88	62.98	0.52	0.44
O:2-CDH-SIDEA-CRM_197_	82.82	36.67	2.26	0.40
O:2-ADH-SIDEA-CRM_197_	51.54	29.56	1.74	0.39
O:2-(EDAC)ADH-CRM_197_	50.19	31.31	1.60	0.52
O:2				0.55
CRM_197_				0.69

**Figure 5:**
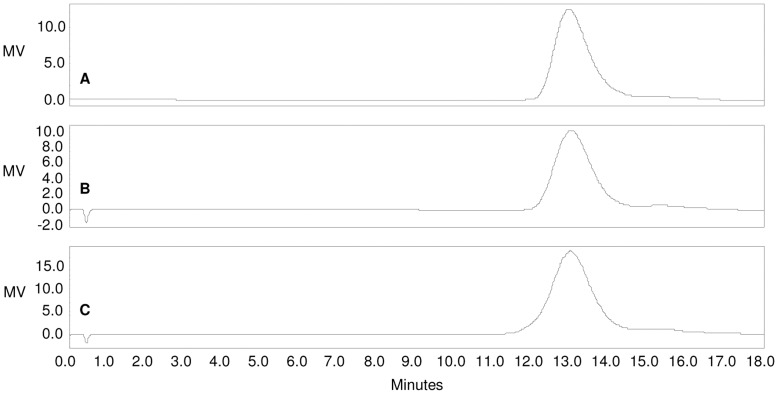
HPLC-SEC analysis of A) O:2; B) O:2-ADH; C) O:2-ADH-SIDEA (RI detection). Samples run on TosoHaas TSK gel 3000 PW_XL_ column; eluent: 0.1 M NaH_2_PO_4_, 0.1 M NaCl, 5% CH_3_CN, pH 7.2; flow rate: 0.5 mL/min. Column void volume: 10.85 min.; total volume: 23.54 min. O:2 molecular weight distribution remains unchanged during the O:2 derivatization steps with ADH and SIDEA (all the samples have Kd of 0.17).

**Figure 6:**
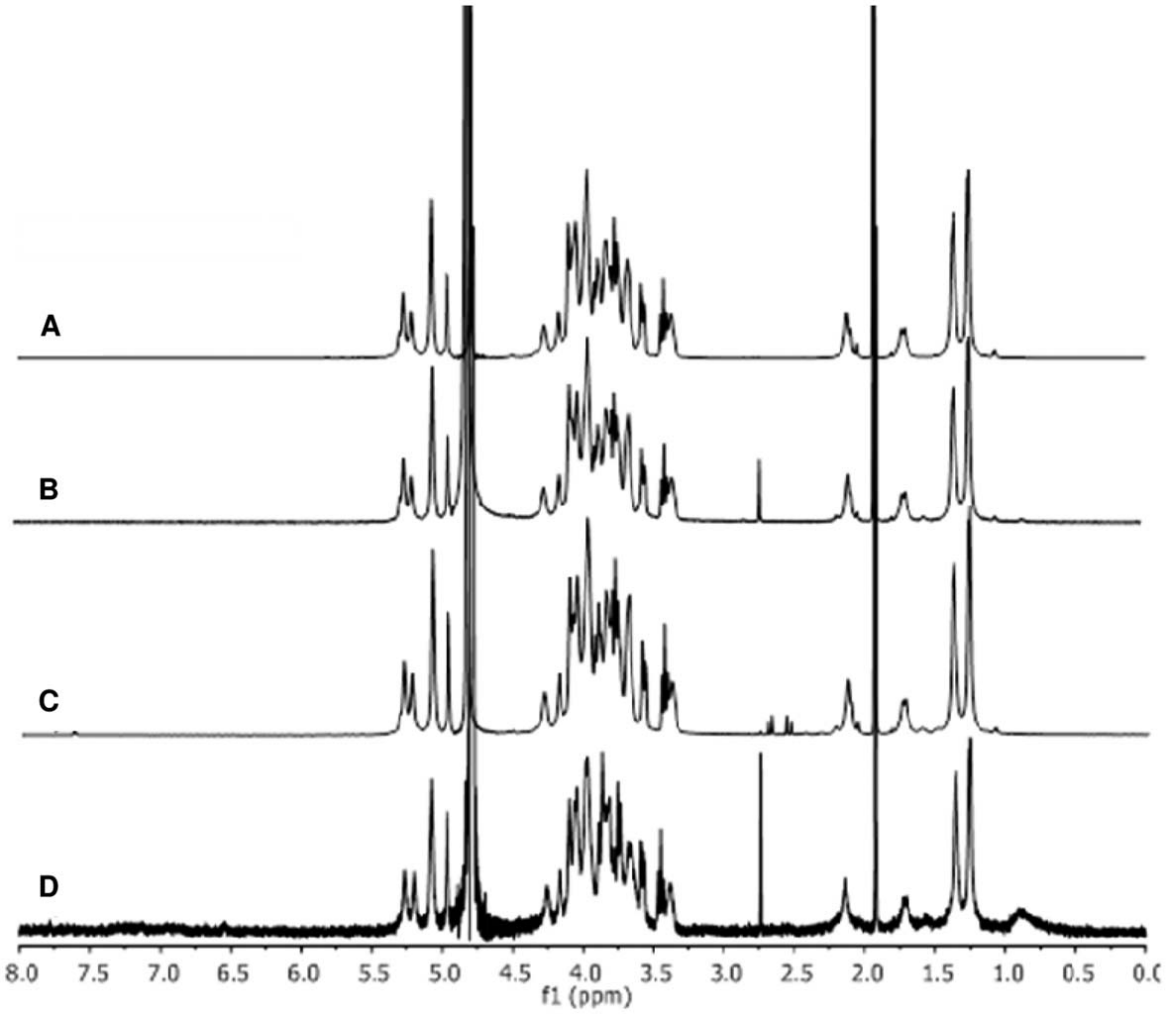
^1^H NMR spectra of A) O:2 (68% OAc); B) O:2-ADH (70% OAc); C) O:2-ADH-SIDEA (68% OAc); D) O:2-ADH-SIDEA-CRM_197_ (67% OAc). O-acetylation level quantified by comparing acetate (released after treatment with NaOD, at 1.91 ppm) and Rha-H6 peaks at 1.40 ppm, and expressed as molar % of O-acetyl with respect to OAg chain repeating units (being Rha present only in the OAg chain, at one sugar per repeating unit). O-acetylation level is maintained at the same level after conjugation.

O:2 molecular weight distribution (detected by HPLC-SEC) remained unchanged during the O:2 activation steps with ADH or CDH, and then with SIDEA (Figure 5). Also, O:2 chain sugar composition was unchanged during conjugation steps as detected by ^1^H NMR and HPAEC-PAD (Table 4, and data not shown for conjugates prepared with CDAP or EDAC). In all conjugates, O-acetylation was maintained at the same level as the underivatized OAg (Figure 6).

**Table 4:** O:2 chain sugar composition remains unchanged during conjugation steps to produce O:2-ADH-SIDEA-CRM_197._

	Sugar composition, molar ratio			
	by ^1^H NMR	by HPAEC-PAD		
Sample	Par/Rha	Man/Rha	Gal/Rha	Glc/Rha
Underivatized O:2	1.06	1.07	1.05	0.74
O:2-ADH	1.08	1.05	0.99	0.73
O:2-ADH-SIDEA	1.08	1.05	0.98	0.83
O:2-ADH-SIDEA-CRM_197_	1.05	1.04	1.07	0.83

**Figure 7:**
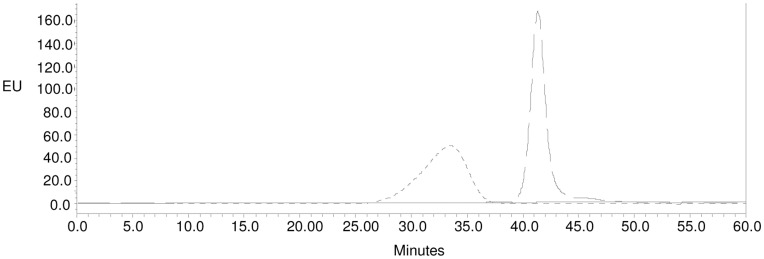
HPLC-SEC profiles (fluorescence emission detection) of O:2-ADH-SIDEA-CRM_197_ (dotted line; 28.7 µg/mL of protein; Kd of 0.39) in comparison to free CRM_197_ (dashed line; 50 mg/mL; Kd of 0.69) and free O:2 (solid line; 1 mg/mL of sugar; Kd of 0.55). 80 µL of each samples run on TosoHaas TSK gel 6000+5000 PW columns; eluent: 0.1 M NaH_2_PO_4_, 0.1 M NaCl, 5% CH_3_CN, pH 7.2; flow rate: 0.5 mL/min. Column void volume: 23.65 min.; total volume: 49.07 min. Free O:2 is not detected by fluorescence emission.

The O:2-ADH-SIDEA-CRM_197_ HPLC-SEC profile detected by fluorescence emission in comparison to free CRM_197_ and free O:2 is shown in Figure 7. Free O:2 is not detected by fluorescence emission and the peak at higher MW of O:2-ADH-SIDEA-CRM_197_ confirmed conjugate formation and the absence of free protein in the purified conjugate.

### Direct Conjugation of O:2 with SIDEA and CRM_197_ (without Derivatization using ADH or CDH)

Conjugation with CRM_197_ occured also after direct OAg derivatization with SIDEA and without previous linkage of ADH or CDH. This is due to the presence of NH_2_ groups on the underivatized OAg, probably as pyrophophoetanolamine (Figure 1). OAg was directly activated with SIDEA, using the same conditions reported for O:2-ADH. Conjugate formation was verified by SDS-PAGE analysis of the conjugation mixture (Figure 3 lane 7) and purified conjugate was similar to the conjugate obtained introducing ADH as linker (Table 5).

**Table 5:** Characterization of conjugates after purification on Sephacryl S-300 HR column.

Conjugate	Total sugar, µg/mL	Total protein, µg/mL	Wt/wt ratio O:2 toCRM_197_	Kd (HPLC-SEC)
O:2-ADH-SIDEA-CRM_197_	51.54	29.56	1.74	0.39
O:2-SIDEA-CRM_197_	108.6	52.8	2.06	0.38
O:2-(TNBS)ADH-SIDEA-CRM_197_	54.8	23.7	2.31	0.44

**Figure 8:**
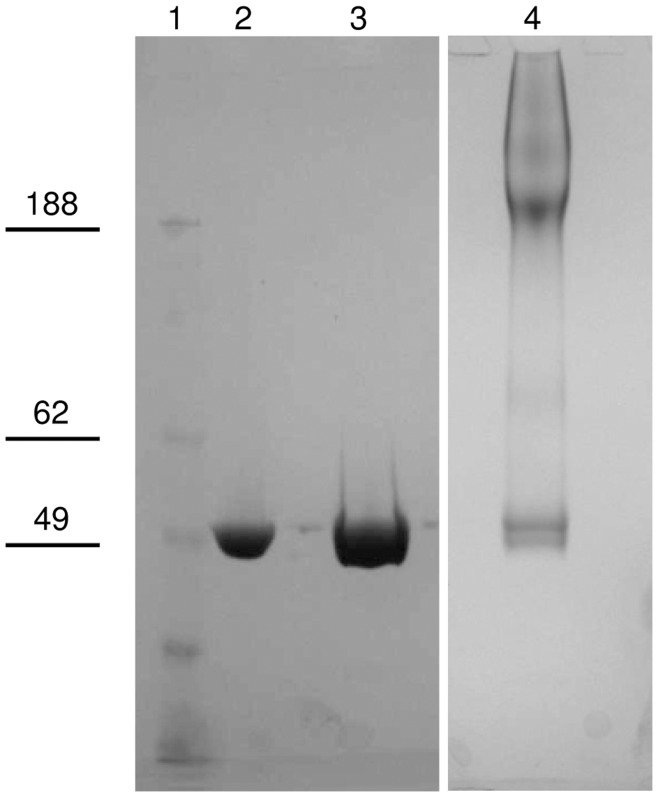
SDS-PAGE analysis. Lane 1: marker, lane 2: CRM_197_ (5 µg), lane 3: conjugation mixture of O:2(TNBS)-SIDEA + CRM_197_ (10 µg of protein), lane 4 conjugation mixture of O:2(TNBS)-ADH-SIDEA + CRM_197_(10 µg of protein).

To verify that conjugate formation was due to the presence of the NH_2_ groups on the OAg, and at the same time to verify that linked ADH was able to bind SIDEA and conjugate to CRM_197_, an O:2 sample was protected with TNBS (same procedure used for the colorimetric method) and tested for conjugation after desalting on G-25 column. The protected OAg was activated with ADH, then with SIDEA and finally conjugated with CRM_197_, obtaining a conjugation product (Table 5). The O:2-(TNBS)ADH-SIDEA-CRM_197_ reaction mixture was also analyzed by SDS-PAGE (Figure 8 Lane 4). In contrast, direct O:2-(TNBS) activation with SIDEA (performed assuming the same NH_2_ groups present before and after reaction with TNBS) and conjugation with CRM_197_ did not work (Figure 8 Lane 3).

### Immunogenicity of Conjugates in Mice

**Figure 9:**
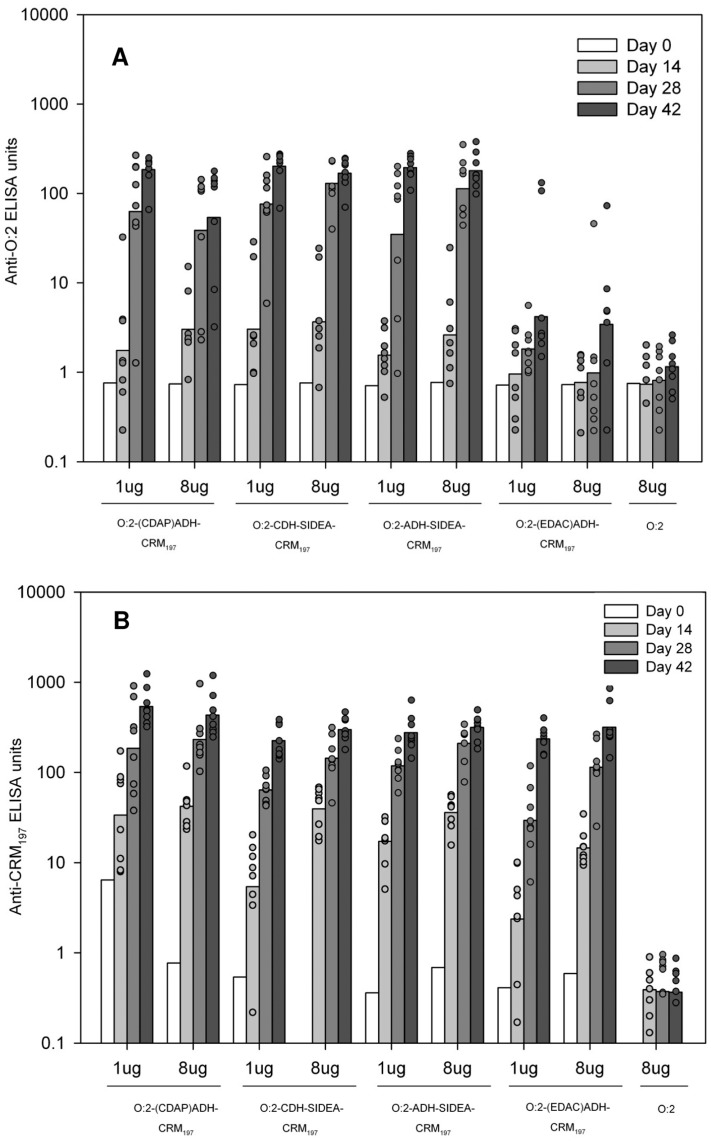
Anti-O:2 IgG (panel A) and Anti-CRM_197_ (panel B) serum IgG ELISA units detected in sera of CD-1 mice immunized with O:2-conjugates and unconjugated O:2. Mice were immunized three times at two week-intervals with the indicated doses of material. Mice were bled and sera collected on the indicated days. Individual animals are represented by the scatter plots; bars represent the group geometric mean.

To assess the ability of conjugates to stimulate anti-OAg antibodies, outbred CD-1 mice were immunized subcutaneously three times at two week-intervals. As shown in Figure 9A, all O:2-CRM_197_ conjugates, except O:2-(EDAC)ADH-CRM_197_, were immunogenic following three immunizations with either 1 µg or 8 µg conjugate. All conjugates, again with the exception of O:2-(EDAC)ADH-CRM_197_ elicited significantly higher anti-O:2 ELISA units than unconjugated O:2 after three immunization with 8 µg (Kruskal Wallis analysis, p<0.001). Subclass antibody analysis showed that anti-O:2 IgG1 was the predominant subclass in sera from animals vaccinated with OAg conjugates (data not shown). This is consistent with a Th2 response raised by a T-dependent antigen. No significant difference in anti-CRM_197_ ELISA units was detected among the conjugates (p<0.001) at either 1 µg/dose or 8 µg/dose on day 42 (Figure 9B). Sera from mice immunized with unconjugated OAg did not produce detectable antibodies to OAg.

### Serum Bactericidal Activity

**Figure 10:**
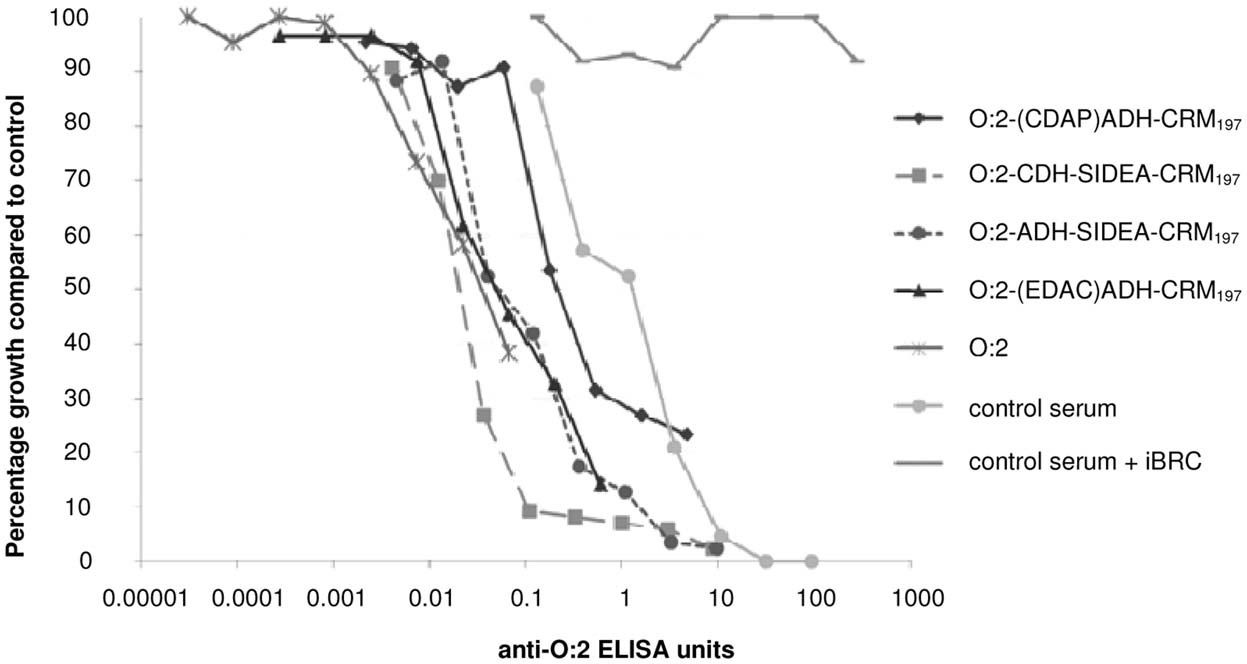
SBA assay performed with pooled mouse sera from different immunization groups and CVD1901. Data are presented as percentage of CFU recovered in test sera with active BRC (and in control serum with inactive BRC) compared with CFU present in negative control, per anti-O:2 ELISA antibody unit of each serum pool.

Serum Bactericidal Activity (SBA) assays were performed using day 42 pooled sera from mice immunized with 8 ug of conjugated or unconjugated O:2 and *S.* Paratyphi A CVD1901 (Figure 10). Inhibition of *S.* Paratyphi A growth in vitro correlated with increasing anti-O:2 ELISA units present in the sera pools. The strongest growth inhibition was observed with those conjugates produced using a selective chemistry; both SIDEA conjugates and O:2-(EDAC)ADH-CRM_197_ resulted in increased inhibition compared to O:2-(CDAP)ADH-CRM_197,_ whose OAg was randomly modified prior to conjugation. Even unconjugated O:2, although far from reaching high levels of bacterial growth inhibition due to the lower amount of antibody present in the sera, presented an inhibition profile similar to the conjugates prepared with unmodified OAg chains. No bacterial growth inhibition was detected using control serum in the presence of iBRC (the same sera in the presence of active BRC is shown as control), indicating a role for complement mediated killing.

## Discussion

Different chemistries were tested for conjugating *S.* Paratyphi A O:2 antigen to CRM_197_. Conjugates obtained by modification of the O-antigen either randomly or specifically through the terminus KDO were compared. Theoretically, conjugation using the KDO would not modify the structure of the O-antigen repeating unit and would lead to the generation of better defined conjugates [36] without formation of crosslinked products. Conjugation procedures previously reported were:

- random activation of -OH groups of the polysaccharide with ADH by CDAP, followed by conjugation with CRM_197_ via carbodiimide chemistry [24]
- activation at the terminus KDO of core region with ADH (using the -COOH group of KDO, by EDAC) followed by conjugation with CRM_197_, always via EDAC chemistry [24], [35].

EDAC chemistry can result in side reactions including cross-linking of the protein (Figure 3). To avoid the possible side reactions, alternative chemistries were studied and a new successful approach was identified (Figure 2). KDO was first activated with ADH through its ketone group by reductive amination, which was followed by a subsequently activation with SIDEA and reaction with CRM_197_. Carbodihydrazide (CDH) was also tried as a shorter linker than ADH, using the same new conjugation protocol. OAg reductive amination using the KDO has been previously described, but much longer reaction times were required (1–2 weeks) [37] than those described here (1 hour). Working at lower pH was essential for the efficiency of this reaction in shorter time (Table 2).

The step of activation with SIDEA was optimized to avoid the use of toxic solvents, such as dioxane or AcOEt, needed to remove free SIDEA and recover the activated OAg. We found that free SIDEA precipitation can be performed adding an aqueous buffer at low pH, making the free linker insoluble and at the same time making slower the hydrolysis of linked active ester groups. Subsequently, O:2-ADH-SIDEA was recovered by precipitation with EtOH. The removal of the free SIDEA worked well, as free active ester groups were less than 10% in moles, and good sugar recoveries were obtained together with high % of NH_2_ groups activation.

Conjugation to CRM_197_ can also be performed without KDO activation by a dihydrazide linker, but through direct activation of the NH_2_ group present on the polysaccharide core region with SIDEA. This chemistry would allow removal of one step in the conjugation process, however, there are concerns related to the stability of the corresponding O:2-SIDEA-CRM_197_ conjugate, as the phosphoester link between the ethanolamine is not as stable as the C-N link introduced by reductive amination. Furthermore, the amount of NH_2_ groups on the underivatized OAg was variable from batch to batch (probably due to the instability of the phosphoester link under the conditions used for OAg purification) and this could be risky in terms of reproducibility of the conjugation process. After the introduction of the more reactive hydrazide linker on the KDO, the reactivity of the amino group on the core region with SIDEA cannot be excluded, with the possibility to have also two active ester groups per OAg chain. Work is ongoing to make the reaction of the hydrazide linker with SIDEA more selective.

Different conjugates were used to vaccinate mice in order to assess their immunogenicity and ability to generate functional antibodies in vitro. All conjugates, with the exception of O:2-(EDAC)ADH-CRM_197_, induced significantly higher levels of anti-O:2 serum IgG antibody responses in comparison to unconjugated O:2. This result confirmed what was already shown by Watson *et al*. that a conjugate obtained by derivatizing the reducing terminus KDO with EDAC and coupling to TT was poorly immunogenic [38]. The new chemistry described here was able to generate good antibodies responses with both the shorter (CDH) and the longer (ADH) linker. These serum antibodies were also shown to have functional activity as demonstrated by their ability to kill *S.* Paratyphi A in vitro. Anti-sera from different conjugates produced similar SBA curves. The greatest bacterial growth inhibition, using sera normalized to anti-O:2 ELISA units, was detected using conjugates generated by selective conjugation chemistries, including the less antigenic O:2-(EDAC)ADH-CRM_197_ (Figure 10). It is possible that the degree of modification of the OAg chain resulting from conjugation may result in anti-O:2 antibodies whose fine specificities influence the bactericidal activity and the overall quality of the immune response induced. Other factors may also be relevant for immunogenicity and SBA, like OAg derivatization levels and the final conjugate structure.

In summary, an amenable conjugate vaccine should be able to elicit an antibody response that is specific, potent and functional. The data presented here encourage the development of an O:2-ADH-SIDEA-CRM_197_ or O:2-CDH-SIDEA-CRM_197_ as promising glycoconjugate vaccines against S. Paratyphi A. Further work is being conducted to verify conjugate stability and process industrialization, with the final aim to combine an O:2 conjugate with Vi-CRM_197_ and produce a bivalent vaccine against enteric fever.
